# Hospital Meetings

**Published:** 1901-06-01

**Authors:** 


### HOSPITAL MEETINGS.

### THE MEDICAL GRADUATES' COLLEGE AND POLYCLINIC.

The festival dinner of the Medical Graduates' College and Polyclinic, Chenies Street, was held last week at the Hotel Cecil, Mr. A. J. Balfour, M.P. (vice-patron), presiding.

After the loyal toasts had been duly honoured,

Mr. Balfour said: It is now my duty to propose the toast of the evening, and I can assure you that I do it with deeper feelings of responsibility than, I think, usually oppress a person in my position, because I know that I am pleading a cause which has not behind it any great wave of popular feeling or emotion; a cause which does not readily or immediately appeal to the man in the street, and yet a cause in which the whole community are deeply and profoundly interested (cheers)—a cause which touches every one of us human beings, and will affect not only our own happiness, our own lives, and the happiness and the lives of these nearest and dearest to us, but the future generations whom it is our business to aid. The toast I have to propose, as you will know, is that of "The Polyclinic." The Polyclinic is in this country as yet a young institution, though well known and fully recognised in almost every other civilised country in the world.

### The Aims of the Polyclinic.

It is an institution with many sides of utility and activity. I will not attempt to dwell upon them all, but there are three which especially appeal to me and which, I venture to think, will especially appeal to you when I attempt to describe them and to tell you in what their chief characteristics consist. The first side of the activity of this institution may be described, perhaps, as directly philanthropic—that is to say, it brings directly home to those men in need of it, and to those who could not otherwise afford it, medical assistance, medical advice, and medical knowledge which would not under other circumstances be at their command. The Polyclinic does something which the great hospitals of this country could not do and do not attempt to do. There are vast sections of our population who can avail themselves, and do avail themselves, of the services of the general practitioner, but who, in cases of extreme difficulty, when their medical attendant would, were they persons of means, recommend them [to go to some expert consulting physician, have not the resources which would enable them to take that course. This is a want not supplied by our hospitals, which cannot be supplied by our hospitals under their existing organisation in any large measure; it is supplied by the Polyclinic. (Cheers). The Polyclinic does give, and hopes to give in ever-increasing measure, to the class of which I speak that expert medical advice which at present is at the command only of the well-to-do. I cannot imagine a charity, in the ordinary sense of the word, which ought more to command the generous support of those who are able to give it. And yet I venture to think that what I may describe as the directly charitable or philanthropic side of this institution is not its most important side. It is not the form of its activity which I think will produce the most far-reaching benefits to mankind, for in addition to this consultative expert medical opinion which it is ready to afford to those who could not otherwise obtain it, the Polyclinic is designed to give opportunities of medical education to those who have, indeed, qualified as medical students, who have gone through the ordinary courses which qualify a man to practise, who are perhaps, in a great, and deservedly great, practice, but who have little opportunity of keeping themselves abreast with the ever-growing mass of medical knowledge. Remember what is the life of a general practitioner in a great practice. I do not believe there is a harder life. (Hear, hear.) I am sure there is no more beneficent life led by any set of men upon the face of this earth. It is a hard day-to-day struggle. (A voice: "And nights.") Ah! A day-to-day and night-to-night struggle with disease (cheers); no certainty of repose, no habitual opportunity of study, constant aid to the poor, to the needy, and to the suffering—aid in many cases but ill-remunerated, aid which calls forth constantly and steadily an amount of unknown and unrecognised self-devotion which, I am sure, must move the heart of anybody who thoroughly realises it. Now, to these hard-worked and overworked general practitioners comes the duty of attempting to make themselves familiar with the latest researches in medical science, the accumulated wealth of medical experience, the vast mass of information contained in medical and other scientific journals concerning the last researches of medical science. How is it to be done? How can it possibly be done under existing conditions? It cannot be done; and the Polyclinic has set itself to work to give to these men, in their rare opportunities of leisure, on the easiest and the cheapest terms, an opportunity of bringing themselves abreast with medical science in its latest development, of coming into personal contact with the leaders of medical thought, and of carying back into their own region of special activity this augmented knowledge which it were hardly possible for them to obtain under the existing conditions of stress and strain in which they live. (Cheers.) Think what this means, not to these medical gentlemen themselves, but to their innumerable patients. Think how much an institution on the lines of the Polyclinic, developed, as I hope to see the Polyclinic develop, may do, not for the education of the medical student, which is amply provided for, but for the education of the medical student after he has become a professional man, when he is, perhaps, as much, or even more, in need of those educational advantages for which at present no machinery exists in these islands. (Hear, hear.) There is yet a third branch of the activity of the Polyclinic to which I would venture to call the attention of this audience, and it is perhaps the branch which we in this country are most apt to neglect.

### The Growth of Medical Knowledge.

I have spoken to you of the growth of medical knowledge, and surely that is one of the most remarkable scientific facts of the last half-century. I do not commit myself to the year, but it is, broadly speaking, in the last half-century that the medical profession have become, not merely admirable masters of the art of healing, but acquainted, not emperically merely, but scientifically, with the causes of many of those maladies with which they have to deal. In reality it is only in the last half of the last half-century that we have realised to the full extent how much these maladies under which mankind groans are due, not to the natural and inevitable decay of the human organism, but to what is literally and absolutely an external parasitic invasion. So far as I am able to judge, that view of a large number of the real scientific causes of so many of the maladies of humanity is not diminishing, but is increasing in its scope; and, while there is, so far as I know, no example in which a disease originally supposed to have a parasitic origin has now been found to have some other origin, every day the leaders of the medical profession are more and more coming to the conclusion that the diseases whose parasitic origin was never even suspected, are, nevertheless, due to invasions of the kind I have endeavoured to describe. (Cheers.) I need not say that in such a company as this I am not going to attempt to discuss the effects which this extraordinary discovery has had upon medical science. I am not going to show how, in the first place, doctors have endeavoured to meet this invasion by a general strengthening of the organism by what are called principles of hygiene, nor how they are prepared to meet it by assisting the natural forces of the body to meet the invader by appropriate medicines, or by such anti-toxins, as serum, that therapy has placed at their disposal, nor how they are even educating the body to meet this invasion by the various forms of vaccination which have been adopted and are from day to day increasing.

### This Country Lagging Behind.

I should be going beyond my depth were I to deal with these important aspects, these vital aspects, these leading aspects of the medical science of to-day, and I have only mentioned them in order to lead up this question, which I put to you in all solemnity and seriousness. Can we honestly say that in this great development of medical knowledge and therapeutic science this country has taken the leading part it ought to have taken? I speak in the presence of gentlemen whose names are of European fame, and who certainly have done their part in the spread of medical knowledge and in the furthering of medical research. I do not forget that in perhaps the two branches of medical advance which have done most to save human life and to diminish human pain—I mean the use of anæsthetics and antiseptic surgical treatment — this country may have a claim to have taken the lead. Happy will be the century on which we are entering if other discoveries are made which will do so much to increase human happiness and to diminish human suffering. And yet, when I have made all allowance for those great claims on the gratitude of the world which I think we possess, it remains the fact that, so far, at least, as I am able to judge, we cannot say that as compared with Germany, or with France, or with Italy we have done all that, perhaps, we might have done as pioneers of medical discovery. I may be wrong. It is only a personal opinion, given for what it is worth, but I fear that any investigator who set himself in a perfectly impartial spirit to examine the respective claims upon the gratitude of mankind of these great nations would not be able, in all honesty and fairness, to say that we had any claim to take the lead. Now, if that is so, do not you think we—the public, the unprofessional and the unscientific public—are in part to blame for that state of things? Do you think that we have shown a recognition of the duties which fall upon us in this matter? We are proud to say that in this country we leave to private enterprise and to private benevolence duties which in other and less fortunate countries are entrusted to the Government. (Cheers.) Yes, that is true; but if that policy is to be successful you must have the private enterprise and the private benevolence, and have we shown the possession of those great qualities in this particular to the extent that we ought to have done? Personally I grieve to say I have no doubt as to the answer that should be given. I do not believe that any man who looks round the equipment of our Universities or medical schools, or other places of education, can honestly say in his heart that we have done enough to equip research with all the costly armoury which research must have in these modern days. (Cheers.) We, the richest country in the world, lag behind Germany, France, Switzerland, and Italy. (Hear, hear.) Is it not disgraceful? (Hear, hear.) Are we too poor ("No!"), or are we too stupid? (Cheers.) Do we lack the imagination required to show what these apparently remote and abstract studies do for the happiness of mankind? We can appreciate that which obviously and directly ministers to human advancement and facility, but seem, somehow or another, to be deficient in that higher form of imagination, in that longer sight, which sees in studies which have no obvious, necessary, or immediate result the foundation of the knowledge which shall give far greater happiness to mankind than any immediate, material, industrial advancement can possibly do; and I fear, and greatly fear, that, lacking that imagination, we have allowed ourselves to lag in the glorious race run now by civilised countries in pursuit of knowledge, and we have permitted ourselves to far too large an extent to depend upon others for those additions to our knowledge which surely we might have made for ourselves. (Cheers.)

### An International Competition.

It is the result of my unfortunate profession—(laughter)—that I am constantly engaged in discussions and conflicts which at the worst have a party significance, but which at the best have but a national significance. But the cause I plead now is not the cause of a party nor of a nation, but the cause of mankind at large. (Cheers.) Every discovery which is made in the laboratory in Germany, France, or Italy is the possession, not of those countries, but of the whole world. Let us not be backward in this great international competition, which surely may be said, in some senses, to balance with that yet more costly and destructive competition in armaments and, it may be, in commerce. Here, at all events, the interests of all nations are at one. Here there should be no undue rivalry, or, if there be, the only rivalry we should permit is what nation should add most to that scientific knowledge on which, on more than the efforts of statesmen, politicians, and soldiers, depends the future progress and happiness of mankind. (Cheers.) These are feelings which I have long entertained, and have taken such opportunities as I could to express, but which, I think, are not sufficiently realised by our fellow-countrymen. I hope that, at all events, the result of this evening's proceedings may be that the Polyclinic, which is devoted to the great cause for which I have endeavoured to plead, may obtain from those who can give it that assistance which is required to make it the useful institution which it potentially is, and only requires your aid fully and perfectly to become. I hope I have not pleaded in vain. (Cheers.) At all events, the cause is one in which I firmly believe; and if this country allows itself to be passed in this race I should regard it as a greater national calamity than a lost market here or some national *contretemps* there, I should feel the higher life of the nation had proved itself inadequate to our national necessities, and that we, who certainly of all nations in the world are able to provide adequate means for our men of science to develop those great capacities which in so many branches of science have made us the leaders of thought for all mankind—that we have been failing shamefully in our duty if we allow ourselves to be surpassed in this particular branch by every civilised nation in the world. I hope I have said enough to justify me in asking you to drink with enthusiasm and subscribe with generosity to the great cause of medical science which I have pleaded to-night. (Cheers.)

The toast was drunk with enthusiasm.

Sir W. Broadbent and Mr. Jonathan Hutchinson responded, and after drinking the toasts of "The Empire," proposed by the Duke of Marlborough, and "The Chairman," by the Bishop of London, the company dispersed.

### BRITISH HOME AND HOSPITAL FOR INCURABLES.

The fortieth annual meeting of the British Home and Hospital for Incurables was held on May 23, Mr. Hampton Hale, the Deputy Chairman, presiding.

From the report and accounts presented it appeared that the income for the year was £15,440, being £700 less than last year. Of this £6,705 was from legacies. Owing to losses by death and other causes annual subscriptions showed a net loss of £12, though £163 had been received in new subscriptions. The expenditure amounted to £15,978, being £538 more than the income. Included in that amount is £1,788 spent on the completion of the new building. The amount paid to pensioners was £6,658, the largest for any one year, and £150 above 1899. The cost of the Home was £230 more, which was accounted for by the fact that the number of inmates has now been increased to 80, the full complement. Office and general expenses show a decrease of £250. The board regretted that they had not only not been able to pay off the debt to the bankers of £2,200, but had been obliged to increase it to the extent of £3,500. During the year the Board of Management had been strengthened by the addition of Colonel Eyre Crabbe, C.B. After 22 years' service Mrs. Taylor had resigned her position as matron, Miss Pierce, the head nurse, being appointed to the vacancy. In consideration of Mrs. Taylor's long and faithful services the Board resolved to grant her an allowance of £2 10s. per month. Gratification was expressed that Queen Alexandra had intimated her intention to continue her patronage of the institution, granted by her as Princess of Wales immediately after her arrival in England in 1861—this being the first charity in which she became interested.

The Chairman moved the adoption of the report and referred to several of the items mentioned in it. He said that the cost per head of their inmates had gone up 14s. 6d., but he thought they need not be frightened at that. It was largely due to the increase in the cost of coal and other items of expenditure. He noticed that a sister charity of similar character spent £62 per head.

The report was adopted, the retiring members of the Board were re-elected, and after some verbal alterations in the bye-laws had been sanctioned votes of thanks brought the proceedings to a close.

